# Multi-Objective Differential Evolution for Automatic Clustering with Application to Micro-Array Data Analysis

**DOI:** 10.3390/s90503981

**Published:** 2009-05-25

**Authors:** Kaushik Suresh, Debarati Kundu, Sayan Ghosh, Swagatam Das, Ajith Abraham, Sang Yong Han

**Affiliations:** 1 Dept. of Electronics and Telecommunication Engg, Jadavpur University, Kolkata, India; E-Mails: kaushik_s1988@yahoo.com; kundu.debarati@gmail.com; sayan88tito@gmail.com; swagatamdas19@yahoo.co.in; 2 Norwegian University of Science and Technology, Norway; E-Mail: ajith.abraham@ieee.org; 3 School of Computer Science and Engineering Chung-Ang University, Seoul, Korea

**Keywords:** differential evolution, multi-objective optimization, fuzzy clustering, micro-array data clustering

## Abstract

This paper applies the Differential Evolution (DE) algorithm to the task of automatic fuzzy clustering in a Multi-objective Optimization (MO) framework. It compares the performances of two multi-objective variants of DE over the fuzzy clustering problem, where two conflicting fuzzy validity indices are simultaneously optimized. The resultant Pareto optimal set of solutions from each algorithm consists of a number of non-dominated solutions, from which the user can choose the most promising ones according to the problem specifications. A real-coded representation of the search variables, accommodating variable number of cluster centers, is used for DE. The performances of the multi-objective DE-variants have also been contrasted to that of two most well-known schemes of MO clustering, namely the Non Dominated Sorting Genetic Algorithm (NSGA II) and Multi-Objective Clustering with an unknown number of Clusters K (MOCK). Experimental results using six artificial and four real life datasets of varying range of complexities indicate that DE holds immense promise as a candidate algorithm for devising MO clustering schemes.

## 1. Introduction

Optimization-based automatic clustering algorithms greatly rely on a cluster validity function (optimization criterion) whose optima appear as proxies for the unknown “correct classification” in a previously unhandled dataset [1]. Different formulations of the clustering problem vary according to the optimization criterion used. Most existing clustering methods, however, attempt to optimize just one such clustering criterion modeled by a single cluster validity index. This often results in considerable observable discrepancies between the solutions produced by different algorithms on the same dataset. A single cluster validity measure is hardly able to judge the correctness of clustering for a wide variety of real life datasets. A wrong choice of the validity measure may lead to poor clustering results. Thus, the single-objective clustering method may prove futile (as judged by means of expert's knowledge) in a context where the criterion employed is inappropriate. In situations where the best solution corresponds to a tradeoff between different conflicting objectives, common sense advocates a multi-objective framework for clustering. In the case of iterative optimization algorithms, it is possible that a single-objective approach might visit such tradeoff solutions during a run, but would not recognize them as good and discard them.

Although there has been a plethora of papers reporting several single-objective evolutionary clustering techniques (a comprehensive survey of which can be found in [1,2]), very little research has been undertaken so far towards the application of evolutionary multi-objective optimization algorithms (EMOA) for pattern clustering [3,4]. A state-of-the-art literature survey indicates that DE has already proved itself as a promising candidate in the field of evolutionary multi-objective optimization (EMO) [5-8]. Earlier it has also been successfully applied to single-objective partitional clustering [9-11].

The work reported in [3] is based on Deb *et al.*'s celebrated NSGA (Non Dominated Sorting genetic Algorithm)-II [12] and the clustering method described in [4] is based on PESA (Pareto Envelope based Selection) II [13]; both algorithms are multi-objective variants of the Genetic Algorithm (GA). However, the multi-objective variants of DE have not been applied to the general data clustering problems till date, to the best of our knowledge. This paper primarily compares the performances of two most representative multi-objective DE algorithms on the multi-objective fuzzy clustering problem. The multi-objective DE-variants considered here are namely the Multi-objective DE (MODE) [6] and DE for Multi-objective Optimization (DEMO) [7] owing to their promising results over many benchmark multi-objective optimization problems. Since DE, by nature, is a real-coded population-based optimization algorithm, we here resort to a centroid-based representation scheme for the search variables. Note that in contrast to single objective optimization that yields a single best solution, in MOO, a number of often conflicting objective functions are optimized simultaneously and thus an MOO algorithm, in general, ends up with a number of Pareto optimal solutions.

None of these Pareto optimal solutions can be improved upon an objective any further without degrading it on another. Here we consider the Xie-Beni index [14] and the Fuzzy C Means (FCM) measure (*J*_q_) [15] as the objective functions. Note that any other and any number of objective functions could be used in the proposed MOO clustering framework. The performance of the multi-objective DE-variants have also been contrasted with two best-known EMOA-based clustering methods to date. The first one of these is MOCK, by Handl and Knowles [4], while the second one is based on NSGA II and was used by Bandyopadhyay *et al.* for pixel clustering in remote sensing satellite image data [3]. Although we experimented with a large variety of datasets, here we report the results for ten representative datasets including some microarray yeast sporulation data [16].

## 2. Multi-Objective Optimization with DE

### 2.1. The MO Problem

In many practical or real life problems, there are many (possibly conflicting) objectives that need to be optimized simultaneously. Under such circumstances there no longer exists a single optimal solution but rather a whole set of possible solutions of equivalent quality. The field of Multi-objective Optimization (MO) [17-19] deals with simultaneous optimization of multiple, possibly competing, objective functions. The MO problems tend to be characterized by a family of alternatives, which must be considered equivalent in the absence of information concerning the relevance of each objective relative to the others.

The family of solutions of an MO problem is composed of the parameter vectors, which cannot be improved in any objective without causing degradation in at least one of the other objectives. This forms the central idea of *Pareto-optimality*. The concepts of *dominance* and *Pareto-optimality* may be presented more formally in the following way [18,19]:

#### Definition 1

Consider without loss of generality the following multi-objective optimization problem with *m* decision variables *x* (parameters) and *n* objectives *y*:

(1) $$\text{Maximize}:\;\vec{Y}=f(\vec{X})=(f_{1}(x_{1},‥‥,x_{m}),‥‥,f_{n}(x_{1},‥‥,x_{m}))$$

where *X⃗* =[*x*_1_, …., *x_m_*]^*T*^ ∈ *p* and *Y⃗*=[*y*_1_,….,*y_m_*]^*T*^ ∈ *O* and where *X⃗* is called decision (parameter) vector, *P* is the parameter space, *Y⃗* is the objective vector, and *O* is the objective space. A decision vector *A⃗* ∈ *P* is said to dominate another decision vector *B⃗* ∈ *p* (also written as *A⃗* ≻ *B⃗*) if and only if:

(2) $$\begin{array}{l} \;\forall i\in \{1,\ldots ,n\}:\;f_{i}(\vec{A})\geq f_{i}(\vec{B})\\ \wedge \exists j\in \{1,\ldots ,n\}:\;f_{j}(\vec{A})>f_{j}(\vec{B}) \end{array}$$

Based on this convention, we can define non-dominated, *Pareto-optimal* solutions as follows:

#### Definition 2

Let *A⃗* ∈ *P* be an arbitrary decision vector.

(a) The decision vector *A⃗ is said to be* non-dominated regarding the set *P'* ⊆ *P* if and only if there is no vector in *P*' which can dominate *A⃗*. Formally,

(3) $$∄\vec{A}\prime \in P\prime :P\prime ≻P$$

(b) The decision (parameter) vector *A⃗* is called Pareto-optimal if and only if *A⃗* is non-dominated regarding the whole parameter space *P*.

### 2.2. The Differential Evolution (DE) Algorithm

DE [20, 21] is a population-based global optimization algorithm that uses a real-coded representation. Its starts with a population of *NP* real-coded search variable vectors initialized randomly in the feasible search space. The *i*-th individual (parameter vector or *chromosome*) of the population at generation (time) *G* is a *D*-dimensional vector containing a set of *D* optimization parameters:

(4) $${\vec{Z}}_{i,G}=[Z_{i,1,G,}Z_{i,2,G},‥‥Z_{i,D,G}]$$

Now, in each generation, a donor *Y⃗_i,G_* is created. The method of creating this donor vector demarcates between the various DE schemes. In one of the earliest variants of DE, now called DE/rand/1 scheme, to create *Y⃗_i,G_* for each *i*-th member, three other parameter vectors (say the *r*_1_, *r*_2_, and *r*_3_-th vectors such that *r*_1_, *r*_2_, *r*_3_ ∈ [1,*NP*] and *r*_1_ ≠ *r*_2_ ≠ *r*_3_ are chosen at random from the current population.

Next the difference of any two of the three vectors is multiplied by a scalar number *F* and the scaled difference is added to the third one, whence we obtain the donor vector *Y⃗_i,G_*. The process for the *j*-th component of the *i*-th vector may be expressed as:

(5) $$Y_{i,j,G}=Z_{i_{1},j,G}+F\cdot (Z_{i_{2},j,G}-Z_{i_{3},j,G})$$

Next a crossover operation takes place to increase the potential diversity of the population. We use ‘binomial’ crossover in which case the number of parameters inherited from the mutant has a (nearly) binomial distribution. Thus for each target vector *Z⃗_i,G_*, a trial vector *R⃗_i,G_* is created in the following fashion:

(6) $$\begin{array}{cc} R_{i,j,G}=Y_{i,j,G}, & \text{if}\;({\text{rand}}_{i,j}\left(0,1\right)\leq \text{Cr}\;\text{or}\;j=j_{\text{rand}})\\ Z_{i,j,G}, & \text{ortherwise} \end{array}$$

for *j* = 1, 2, ….., *D* and *rand_j_* (0, 1) ∈[0,1] is the *j*-th evaluation of a uniform random number generator. *j_rand_* ∈ [0,1,….,*D*] is a randomly chosen index which ensures that *R⃗_i,G_* gets at least one component from *Y⃗_i,G_*. To keep the population size constant over subsequent generations, the next step of the algorithm calls for ‘selection’ in order to determine which one between the target vector and trial vector will survive in the next generation i.e. at the next generation *G* = *G*+1. If the trial vector yields a better value of the fitness function, it replaces its target vector in the next generation; otherwise the parent is retained in the population:

(7) $$\begin{array}{cc} {\vec{Z}}_{i,G+1}={\vec{R}}_{i,G} & \text{if}\;f({\vec{R}}_{i,G})\leq f({\vec{Z}}_{i,G})\\ ={\vec{Z}}_{i,G} & \text{if}\;f({\vec{R}}_{i,G})>f({\vec{Z}}_{i,G}) \end{array}\}$$

where *f*(.) is the function to be minimized.

### 2.3. The Multi-Objective Variants of DE

We consider here the two most promising multi-objective variants of DE: the Multi-Objective DE (MODE) [6] and the DE for Multi-objective Optimization (DEMO) [7]. We briefly discuss here the outline of the algorithms instead of reiterating their details, already available in the cited literature.

#### 1) MODE

MODE was proposed by Xue *et al.* in [6]. This algorithm uses a variant of the original DE, in which the best individual is adopted to create the offspring. A Pareto-based approach is introduced to implement the selection of the best individual. If a solution is dominated, a set of non-dominated individuals can be identified and the “best” turns out to be any individual (randomly picked) from this set. Also, the authors adopt (μ + λ) selection, Pareto ranking and crowding distance in order to produce and maintain well-distributed solutions. Xue *et al.* used MODE to solve five high-dimensional unconstrained problems with 250,000 evaluations and the results are compared only to those obtained by SPEA [19].

#### 2) DEMO

DEMO was proposed by Robic and Filipic [7]. This algorithm combines the advantages of DE with the mechanisms of Pareto-based ranking and crowding distance sorting. DEMO only maintains one population and it is extended when newly created candidates take part immediately in the creation of the subsequent candidates. This enables a fast convergence towards the true Pareto front, while the use of non-dominated sorting and crowding distance (derived from the NSGA-II [1]) of the extended population promotes the uniform spread of solutions. DEMO is implemented in three variants (DEMO/parent, DEMO/closest/dec and DEMO/closest/obj) [7]. Below we provide a pseudo-code for MODE/parent:

1. Evaluate the initial population ***P*** of random individuals.
2. While stopping criterion not met, do:
  2.1. For each individual *X⃗_i_* (i = 1 … *NP*) from ***P*** repeat:
  (a) Create candidate *U⃗_i_* from parent *X⃗_i_*.
  (b) Evaluate the candidate.
  (c) If the candidate dominates the parent, the candidate replaces the parent.
  If the parent dominates the candidate, the candidate is discarded.
  Otherwise, the candidate is added in the population.
  2.2. If the population has more than population size *NP* individuals, truncate it.
  2.3. Randomly enumerate the individuals in ***P***.

In DEMO the candidate replaces the parent if it dominates it. If the parent dominates the candidate, the candidate is discarded. Otherwise (when the candidate and the parent is non-dominated with regard to each other), the candidate is added to the population. This step is repeated until *NP* number of candidates is created. After that, we get a population of the size between *NP* and 2.*NP*. If the population has enlarged, we have to truncate it to prepare it for the next step of the algorithm.

The truncation consists of sorting the individuals with non-dominated sorting and then evaluating the individuals of the same front with the crowding distance metric. The truncation procedure keeps in the population only the best *NP* individuals (with regard to these two metrics). The described truncation is derived from NSGA-II. DEMO incorporates two crucial mechanisms. The immediate replacement of the parent individual with the candidate that dominates it is the core of DEMO. The newly created candidates that enter the population (either by replacement or by addition) instantly take part in the creation of the following candidates. This emphasizes elitism within reproduction, which helps achieving the first goal of multi objective optimization – convergence to the true Pareto front. The second mechanism is the use of non-dominated sorting and crowding distance metric in truncation of the extended population. Besides preserving elitism, this mechanism stimulates the uniform spread of solutions. This is needed to achieve the second goal – finding as diverse non-dominated solutions as possible. DEMO's selection scheme thus efficiently pursues both goals of multi objective optimization.

The other two variants were inspired by the concept of Crowding DE as recently introduced by Thomsen [22]. When optimizing functions with many optima, we would sometimes like not only to find one optimal point, but also discover and maintain multiple optima in a single algorithm run. For this purpose, Crowding DE can be used. Crowding DE is basically conventional DE with one important difference. Usually, the candidate is compared to its parent. In Crowding DE, the candidate is compared to the most similar individual in the population. The applied similarity measure is the Euclidean distance between the two solutions.

The second, DEMO/closest/dec, works in the same way as DEMO/parent, with the exception that the candidate solution is compared to the most similar individual in decision space. If it dominates it, the candidate replaces this individual; otherwise it is treated in the same way as in DEMO/parent. The applied similarity measure is the Euclidean distance between the two solutions in decision space. In the third variant, EMO/closest/obj, the candidate is compared to the most similar individual in objective space. DEMO/closest/dec and DEMO/closest/obj need more time for one step of the procedure than DEMO/parent. This is because at every step they have to search for the most similar individual in the decision and objective space, respectively.

## 3. Multi-Objective Clustering Scheme

### 3.1. Search-Variable Representation and Scheme for Finding Correct Number of Clusters

In the proposed method, for *n* data points, each *d*-dimensional, and for a user-specified maximum number of clusters, *K*_max_ a chromosome is a vector of real numbers of dimension *K*_max_ + *K*_max_ × *d*. The first *K*_max_ entries are positive real numbers in (0, 1], each of which controls whether the corresponding cluster is to be activated (i.e. to be really used for classifying the data) or not. The remaining entries are reserved for *K*_max_ cluster centers, each *d*-dimensional. For example, the *i*-th vector is represented as:

(8) $${\vec{Z}}_{i,G}=\begin{array}{cccccccc} T_{i,1} & T_{i,2} & \ldots ‥ & T_{i,K_{\max}} & {\vec{m}}_{i,1} & {\vec{m}}_{i,2} & \ldots \ldots & {\vec{m}}_{i,k_{\max}} \end{array}$$

The *j*-th cluster center in the *i*-th chromosome is active or selected for partitioning the associated dataset if *T_i,j_* >0.5. On the other hand, if *T_i,j_* >0.5, the particular *j*-th cluster is inactive in the *i*-th vector in DE population. Thus the *T_i,j_* s behave like control genes (we call them *activation thresholds*) in the vector governing the selection of the active cluster centers. The rule for selecting the actual number of clusters specified by one vector is:

(9) $$\text{if}\;T_{i,j}>0.5\;\text{THEN}\;\text{the}\;j‐\text{th cluster center}\;{\vec{m}}_{i,j}\;\text{is ACTIVE ELSE}\;{\vec{m}}_{i,j}\;\text{is INACTIVE}.$$

### 3.2. Selecting the Objective Functions

The performance of a multi-objective clustering algorithm critically depends upon the clustering objectives it tries to optimize simultaneously. Conflict among the objective functions is often beneficial since it guides to globally optimal solutions. It also ensures that no single clustering objective is optimized leaving other probable significant objectives unnoticed.

In this work we choose the Xie-Beni index *XB_q_* and a penalized version of the FCM function *J_q_* as the two objectives. The FCM measure *J_q_* may be defined as:

(10) $$J_{q}=(1+k)\sum_{j=1}^{n}\sum_{i=1}^{k}u\frac{q}{ij}\cdot d^{2}({\vec{Z}}_{j},{\vec{m}}_{i}),\;1\leq q\leq \infty$$

where *q* is the fuzzy exponent, *d* indicates a distance measure between the *j*-th pattern vector and *i*-th cluster centroid, *k* is the number of active cluster centroids and *u_ij_* denotes the membership of *j*-th pattern in the *i*-th cluster. The XB index is defined as a function of the ratio of the total variation σ to the minimum separation sep of the clusters. Here σ and *sep* may be written as:

(11) $$\sigma =\sum_{p=1}^{n}\sum_{i=1}^{k}u_{ip}^{2}\cdot d({\vec{m}}_{i},{\vec{Z}}_{p})$$

(12) $$\text{and}\;\text{sep}(Z)=\underset{i\neq j}{\min}\{d^{2}({\vec{m}}_{i},{\vec{m}}_{j})\}$$

The XB index is then written as:

(13) $$XB_{q}=\frac{\sigma }{n\times \text{sep}(Z)}=\frac{\sum_{p=1}^{n}\sum_{i=1}^{k}u_{ip}^{2}\cdot d({\vec{m}}_{i},{\vec{Z}}_{p})}{n\times \min_{i\neq j}\left\{d^{2}({\vec{Z}}_{i},{\vec{Z}}_{j})\right\}}$$

Note that when the partitioning is compact and the individual clusters are well separated, value of σ should be low while *sep* should be high, thereby yielding lower values of *XB_q_* index. The objective therefore is to minimize the XB index. For computing the measures described in equations (10) and (13), the centers encoded in a DE vector are first extracted. Let the set of centers be denoted by {*m⃗*_1_, *m⃗*_2_,…, *m⃗_k_*}. The membership value of the *j*-th pattern in *i*-th cluster *u_ij_* = 1,2,….*k* and *j* = 1,2,…., *n* are computed as:

(14) $$u_{ij}=\frac{1}{\sum_{p=1}^{k}{\left(\frac{d({\vec{m}}_{i},{\vec{Z}}_{j})}{d({\vec{m}}_{p},{\vec{Z}}_{j})}\right)}^{\frac{2}{q-1}}}$$

**Figure 1.**
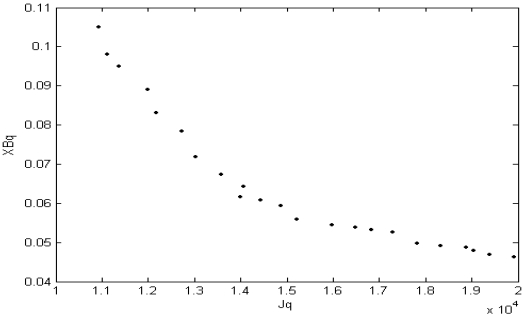
Non-dominated Pareto front for artificial dataset_3.

Note that while computing the *u_ij_* s, using equation (12), if *d* (*m⃗_p_, Z⃗_j_*) is equal to zero for some *p*, then *u*_ij_ is set to zero for all *i* =1,2,…. *k, i* ≠ *j*, while *u_pj_* is set equal to one. Subsequently the centers encoded in a vector are updated using the following assignment:

(15) $${\vec{m}}_{p}=\frac{\sum_{j=1}^{n}{\left(u_{pj}\right)}^{q}\cdot {\vec{Z}}_{j}}{\sum_{j=1}^{n}{\left(u_{pj}\right)}^{q}}$$

and the cluster membership values are recomputed. Note that the *XB_q_* index is a combination of global (numerator) and particular (denominator) situations. The numerator is similar to *J_m_* but the denominator has a factor that gives the separation between to minimum distant clusters. Hence this factor only considers the worst case, i.e. which two clusters are closest to each other and forgets about the other partitions. Here, greater value of the denominator (lower value of whole index) signifies a better partitioning. Thus it is evident that *J_q_* and *XB_q_* indices should be simultaneously minimized in order to get good solutions. The two terms at the numerator and the denominator of *XB_q_* may not attain their best values for the same partitioning when the data has complex and overlapping clusters, such as remote sensing image and micro-array data. Figure 1 shows, just for the sake of illustration, the final Pareto-optimal front (composed of non-dominated solutions) of one of the runs of the MODE algorithm for the artificial dataset_3 (described in the next section), to demonstrate the contradictory nature of *J_q_* and XB indices.

Note that except MOCK, all the DE-based algorithms here use the objective functions described in (10) and (13). The NSGA-II based algorithm described in [3] use a plain FCM index that incorporates no compensation due to large number of clusters. This is obvious, as the method of [3] assumes the number of clusters to be known beforehand, whereas, the multi-objective clustering framework proposed here makes room for a variable number of clusters and the modified FCM index of (10) penalties a large number of clusters. MOCK also uses two conflicting objective functions known as the *overall deviation* and *connectivity*. The overall deviation is computed as the overall summed distances between data items and their corresponding cluster center:

(16) $$\text{Dev}(C)=\sum_{C_{k}\in C}\sum_{i\in C_{k}}\delta (i,{\vec{m}}_{k})$$

where C is the set of all clusters, is the centroid of cluster, and δ is the chosen distance function (here, the Euclidean distance). As an objective, overall deviation should be minimized.

Again connectedness evaluates the degree to which neighboring data points have been placed in the same cluster. It is computed in the following way:

(17) $$\text{Conn}(C)=\sum_{i=1}^{n}\left(\sum_{i=1}^{n}x_{i,nn_{ij}}\right)$$

where $x_{r,s}=\overset{1}{\;}/\underset{j}{\;}$, if there does not exist any *C_k_* such that *r* ∈ *C_k_* ∩ s ∈ *C_k_*,

*x_r,s_* = 0, otherwise.

*nn_ij_* is the *j*-th nearest neighbor of datum *i, n* is the size of the clustered data set, and *L* is a parameter determining the number of neighbors that contribute to the connectivity measure. As an objective, connectivity should be minimized.

### 3.3. Avoiding Erroneous Vectors

There is a possibility that in our scheme, during computation of the *XB* or *J_q_*, a division by zero may be encountered. This may occur when one of the selected cluster centers in a DE-vector is outside the boundary of distributions of the data set. To avoid this problem we first check to see if any cluster has fewer than two data points in it. If so, the cluster center positions of this special chromosome are re-initialized by an average computation. We put $\overset{n}{\;}/\underset{k}{\;}$ data points for every individual cluster center, such that a data point goes with a center that is nearest to it.

### 3.4. Selecting the Best Solution from Pareto-Front

Multi-objective clustering does not return a single solution, but a set of clustering solutions. These individual groupings correspond to different tradeoffs between the two objectives and, in our case, also consist of different numbers of clusters. Several researchers have already investigated the identification of promising solutions from Pareto front approximations recently [23, 24]. These works have primarily dealt with the reduction of the size of the approximation set in absence of additional expert's knowledge. For choosing the most interesting solutions from the Pareto front, we follow a similar technique as the one used in MOCK. It is inspired by Tibshirani *et al.*'s Gap statistic [25], a statistical method to determine the number of clusters in a data set. The Gap statistic is based on the expectation that the most suitable number of clusters shows in a significant “knee” when plotting the performance of a clustering algorithm (in terms of a selected internal evaluation measure) as a function of the number of clusters. We use the same heuristic technique described in pages 65 – 66 of [4] to generate the attainment scores for each clustering problem. Finally, we plot the attainment scores as a function of the number of clusters. All solutions corresponding to the local optima in the resulting plot are considered as promising solutions. The global maximum in this plot may be considered as the estimated “best” solution.

### 3.5. Evaluating the Clustering Quality

In this work, the final clustering quality is evaluated using two external measures. Specifically we choose the adjusted Rand index [26] (which is a generalization of the Rand index [27]) and the sihouette index [28]. Mostly we use the adjusted Rand index for evaluating the quality of partitioning in those 9 datasets for which the nominal classification is known. Silhouette index is used for the Yeast microarray dataset, corresponding to which no standard or nominal classification exists. In most recent and existing literatures, like [3, 29], the clustering quality on yeast sporulation data has been judged by using this index.

The adjusted Rand index comes as a generalization of the Rand Index [27]. It introduces a statistically induced normalization in order to yield values close to 0 for random partitions. Using a representation based on contingency tables, the Adjusted Rand Index is given by:

(18) $$R=\frac{\sum_{i,j}(\begin{array}{c} n_{ij}\\ 2 \end{array})-[\sum_{i}(\begin{array}{c} n_{i+}\\ 2 \end{array})\cdot \sum_{i}\left(\begin{array}{c} n_{+j}\\ 2 \end{array})]/(\begin{array}{c} n\\ 2 \end{array}\right)}{\frac{1}{2}[\sum_{i}(\begin{array}{c} n_{i+}\\ 2 \end{array})+\sum_{j}(\begin{array}{c} n_{+j}\\ 2 \end{array})]-[\sum_{i}(\begin{array}{c} n_{i+}\\ 2 \end{array})\cdot \sum_{j}\left(\begin{array}{c} n_{+j}\\ 2 \end{array})]/(\begin{array}{c} n\\ 2 \end{array}\right)},$$

where *n* is the total number of data points, and *n_ij_* is the number of data points classified into class *i* in the experimental classification and into class *j* in the real classification. Also *n*_*i*+_ = Σ_*j*_ *n_ij_* is the number of objects classified into cluster *i* in the experiment, and *n*_+*j*_ = Σ_*i*_ *n_ij_* is the number of objects classified into class *j* in the actually known classification.

Silhouette width reflects the compactness and separation of the clusters. Given a set of data points *Z*= {*Z⃗*_1_, …, *Z⃗_n_*} and a given clustering solution *C* = {*C*_1_, *C*_2_,…,*C_k_*}, the Silhouette width *s*(*Z⃗_j_*) for each data *Z⃗_j_* belonging to cluster *C_i_* indicates a measure of the confidence of belongingness, and it is defined as:

(19) $$s({\vec{Z}}_{j})=\frac{b({\vec{Z}}_{j})-a({\vec{Z}}_{j})}{\max(a({\vec{Z}}_{j}),b({\vec{Z}}_{j}))}.$$

Here *a*(*Z⃗_j_*) denotes the average distance of data point *Z⃗_j_* from the other data points of the cluster to which the data point *Z⃗_j_* is assigned (i. e. cluster *C_i_*). On the other hand, *b*(*Z⃗_j_*) represents the minimum of the average distances of data point *Z⃗_j_* from the data points belonging to clusters *C_r_, r* = 1,2,…,*k* and *r* ≠ *i*. The value of *s*(*Z⃗_j_*) lies between -1 and +1. Large values of *s*(*Z⃗_j_*) (near to 1) indicate that the data point *Z⃗_j_* is well clustered. Value of *s*(*Z⃗_j_*) around 0 means that the data point lies between two clusters and a negative value of *s*(*Z⃗_j_*) indicates that the data point *Z⃗_j_* is probably placed in a wrong cluster. Overall Silhouette index *s*(*C*) of a clustering solution *C* = {*C*_1_, *C*_2_,…,*C_k_*} is defined as the mean Silhouette width over all the data points:

(20) $$s(C)=\frac{1}{n}\sum_{j=1}^{n}s({\vec{Z}}_{j})$$

Greater values of s(*C*) (near to 1) reflect that most of the data points are correctly clustered and this in turn indicates a better clustering solution. Silhouette index can be evaluated for any distance measure.

### 3.6. Putting It Together

Putting the above procedures together, we may now give an over all pseudo-code of the DE-based multi-objective clustering algorithm in the following way:

#### Pseudo code of clustering with multi-objective DE

1. Randomly initialize the control genes and cluster centroids for the maximum number of clusters for the initial population. Each control gene corresponds to a cluster centroid. A centroid is said to be “active” if the corresponding gene exceeds 0.5.
2. While stopping criterion not met, do:
  2.1. Evaluate values of Xie-Beni and penalized FCM indices.
  2.2. Create trial vector of control genes and cluster centroids using standard DE operators and optimize the indices using multi-objective differential evolution. The control genes and cluster centroids are thus evolved.
3. Find the Pareto front of the final set of solutions and find the best solution using gap statistic. The solution at knee point on the Pareto front corresponds to the correct number of clusters.

## 4. Experimental Results

### 4.1. Datasets Used

The experimental results showing the effectiveness of multi-objective DE based clustering has been provided for six artificial and four real life datasets. The artificial datasets are named as Dataset_1 to Dataset_6, with number of clusters varying from 3 to 10. Table 1 presents the number of objects, dimensionality and the number of clusters for each data. The real-life datasets are iris, wine, breast-cancer [30] and the yeast sporulation data. We consider here the microarray data on the transcriptional program of sporulation in budding yeast, the collection and analysis of which have been described in [16]. The sporulation dataset is publicly available from the website: http://cmgm.stanford.edu/pbrown/ sporulation. This dataset consists of 6,118 genes measured across seven time points (0, 0.5, 2, 5, 7, 9 and 11.5 h) during the sporulation process of budding yeast. The data are then log-transformed. Among the 6,118 genes, those whose expression levels did not change significantly during the harvesting, have been ignored from further analysis. This is determined with a threshold level of 1.6 for the root mean squares of the log2-transformed ratios. The resulting set consists of 474 genes. Please note that for the yeast sporulation dataset, we have used the Pearson correlation coefficient based distance measure [31], instead of the conventional Euclidean distance (which has been used for the rest of the datasets), as it has been shown to be more effective for clustering microarray datasets [32].

### 4.2. Other Competitor Algorithms

This paper compares the clustering performances of two promising multi-objective DE-variants with two other evolutionary multi-objective clustering techniques: the NSGA – II [23] and MOCK [24]. Below we briefly describe these techniques, to provide an idea of their conceptual difference with the DE-based MO clustering algorithms.

#### 1) The NSGA II based Clustering Algorithm

Bandyopadhyay *et al.* [3] proposed a non-automatic multi-objective scheme for clustering the pixels of remote sensing satellite images into several fuzzy partitions. They employed the NSGA II algorithm to optimize a number of fuzzy cluster validity indices simultaneously. In NSGA II, initially a random parent population *G*_0_ of size *N* is created. Then the population is sorted based on the non-domination relation. Each solution of the population is assigned a fitness that is equal to its non-domination level. A child population *H*_0_ is created from the parent population *G*_0_ by using binary tournament selection, recombination, and mutation operators. Generally according to this algorithm, initially a combined population *R_t_* = *G_t_* + *H_t_* is formed of size *R_t_*, which is 2*N*. Now all the solutions of *R_t_* are sorted based on their non-domination status. If the total number of solutions belonging to the best non-dominated set *F*_1_ is smaller than *N, F*_1_ is completely included into *G_t+_*_1_. The remaining members of the population *G_t+_*_1_ are chosen from subsequent non-dominated fronts in the order of their ranking. To choose exactly *N* solutions, the solutions of the last included front are sorted using the crowded comparison operator and the best among them (i.e., those with larger values of the crowding distance) are selected to fill in the available slots in *G_t+_*_1_. The new population *G_t+_*_1_ is now used for selection, crossover, and mutation to create a new population *H_t+_*_1_ of size *N* and the process continues. The crowding distance operator is also used in the parent selection phase in order to break a tie in the binary tournament selection. This operator is instrumental in maintaining diversity in the Pareto front.

The resultant set of near-Pareto-optimal solutions contained a number of non-dominated solutions, which the user could judge relatively and pick up the most promising one according to the problem requirements. Real-coded encoding of the cluster centers was used for this purpose. We shall use this algorithm for clustering synthetic as well as real life datasets with real numerical attributes in this paper.

#### 2) The MOCK Algorithm

Handl and Knowles proposed a multi-objective clustering scheme known as Voronoi Initialized Evolutionary Nearest-Neighbor Algorithm (VIENNA [32]), which is based on PESA II [33] and simultaneously optimizes two objectives. It employs a straightforward encoding of a clustering, with a gene for each data item and its allele value specifying the cluster to which the data item should belong. VIENNA needed an advanced initialization scheme based on Voronoi cells and directed mutation to make up for deficiencies in its encoding. In addition, it is non-automatic and does not provide any means to select good solutions from the final Pareto front. Handl and Knowles [4, 34] proposed an improved EMO-based clustering algorithm, which they named M*ulti-Objective Clustering with Automatic k Determination* (MOCK). They fine-tuned one of the objectives used in VIENNA and found a better encoding that does not fix the number of clusters and because of good locality and heritability, allows a much more effective exploration of the search space via suitable operators. They also developed a method for selection of best solutions from the Pareto front based on a null model, thus also determining the number of clusters, automatically. MOCK was also extended in [35] for improving its scalability to large, high-dimensional datasets and data with large number of clusters. Handle and Knowles also introduced MOCK-around-medoids, which allows for the clustering of similarity data [36] (as opposed to vectorial data, i.e. points in a metric space). Here we shall use for comparison the version of the algorithm described in [4].

### 4.3. Parameters for the Algorithms

**Figure 2.**
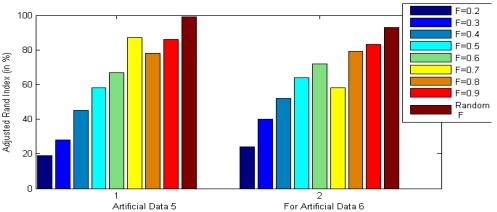
Final clustering result for artificial datasets 5 and 6 with MODE for different settings of scale factor *F*.

**Figure 3.**
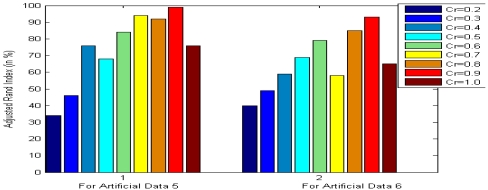
Final clustering result for artificial datasets 5 and 6 with MODE for different settings of crossover rate *Cr*.

All the multi-objective DE variants have been used with 40 parameter vectors in each generation and each run of each algorithm was continued for 100 generations. The value of scale factor *F* is a random value between 0.5 and 1 and *Cr* was fixed at 0.9. These parameter values have been recommended for DE after performing a series of hand-tuning experiments. First we use standard values of *F* (0.8) and *Cr* (0.9) [21] and repeat the clustering techniques on various datasets with varying population size *NP*. We find that keeping *NP* around 40 gives reasonable computational time over a wide range of datasets. Next, fixing *NP* at 40 we varied *F* and *Cr* respectively and obtained the clustering results on several datasets in terms of the adjusted Rand index. Figure 2 shows a glimpse of these experiments with MODE on artificial datasets 5 and 6 for various values of *F*, keeping *Cr* at 0.9. In each case we report the average adjusted Rand index for 30 independent runs of the algorithms. Similarly Figure 3 presents the final accuracy of MODE on the same two datasets for various values of *Cr* keeping the value of *F* random between 0.5 and 1.5. It is evident from both the figures that the suggested parameter setting (*F* random and *Cr* = 0.9) gives best clustering performance with the multi-objective DE variants. We do not provide the results for all the available datasets to save space and considering the fact that they show more or less similar trend as shown in Figures 2 and 3.

The other parameters for the multi-objective GA (NSGA II) based clustering are fixed as follows: number of generations = 100, population size = 50, crossover probability = 0.8, $\text{mutation probability}=\frac{1}{\text{Chromosome}\_\text{length}}$. Please note that the two DE variants and the NSGA II use the same parameter representation scheme. Clustering with MOCK was performed with the source codes available from http://dbkgroup.org/handl/mock/.

### 4.4. Presentation of Results

The mean adjusted Rand index values of the best-of-run solutions provided by six contestant algorithms over the nine datasets (for which the nominal classifications are already known) have been provided in Table 2. The best entries have been marked in boldface in each row. Note that for the DE-based MO clustering techniques, the number of estimated classes correspond to the best solution from the Pareto optimal front chosen by using the technique described in Section 3.4. Table 3 shows the Silhouette index values for yeast sporulation data as no standard nominal classification is known for this dataset. Note that we have not provided the results for other datasets in terms of Silhouette index as analysis based on the Silhouette width is not an objective evaluation technique, as it may be biased towards algorithms optimizing objectives related to the Silhouette width.

Tables 4 and 5 show the results of unpaired *t* tests (standard error of difference of the two means, 95% confidence interval of this difference, the *t* value, and the two-tailed *P* value) between the best and second best algorithms in terms of both average adjusted Rand index and Silhouette index. For all cases in Tables 4 and 5, sample size = 30 and number of degrees of freedom = 58. Here all the *t*-tests have been performed using the statistical calculator available from the website: http://www.graphpad.com/quickcalcs/ttest1.cfm

**Figure 4.**
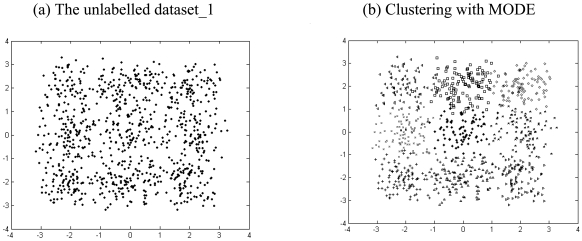
Clustering result for artificial dataset_1.

**Figure 5.**
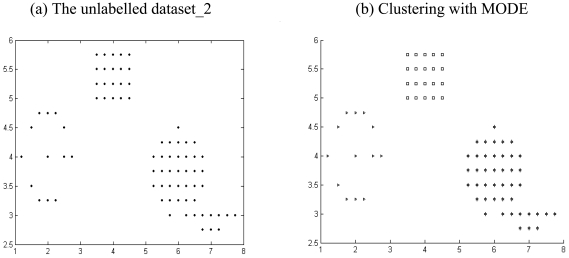
Clustering result for artificial dataset_2.

**Figure 6.**
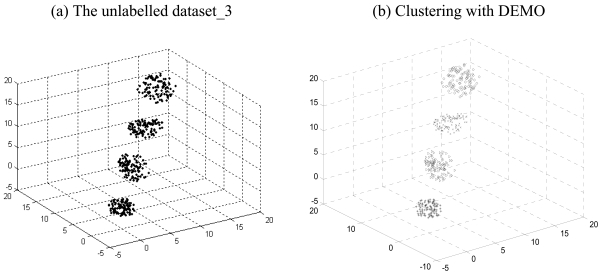
Clustering result for artificial dataset_3.

**Figure 7.**
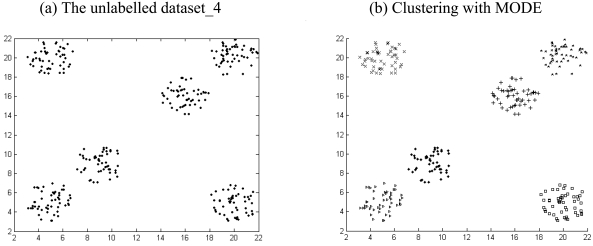
Clustering result for artificial dataset_4.

**Figure 8.**
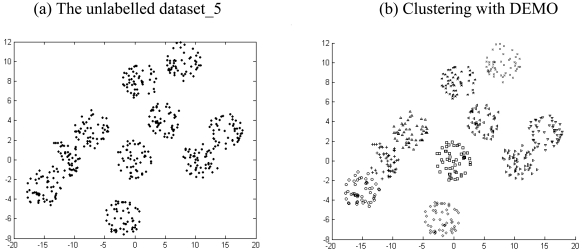
Clustering result for artificial dataset_5.

**Figure 9.**
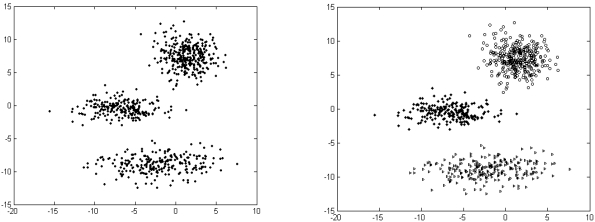
Clustering result for artificial dataset_6.

The results listed in Tables 2 to 4 indicate that there is always one or more multi-objective DE variant that beats the NSGA II or MOCK in terms of mean Silhouette index and adjusted Rand index in a statistically significant fashion. The six unlabelled artificial datasets and the corresponding clustered data with the best performing algorithm (which happens to be one of the two multi-objective DE variants) have been depicted in Figures 4 to 9.

### 4.5. Significance and Validation of Microarray Data Clustering Results

**Figure 10.**
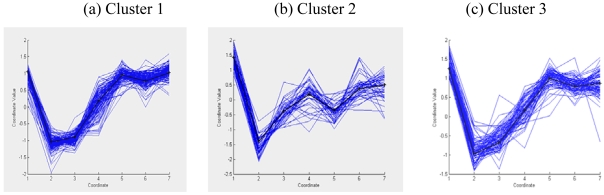

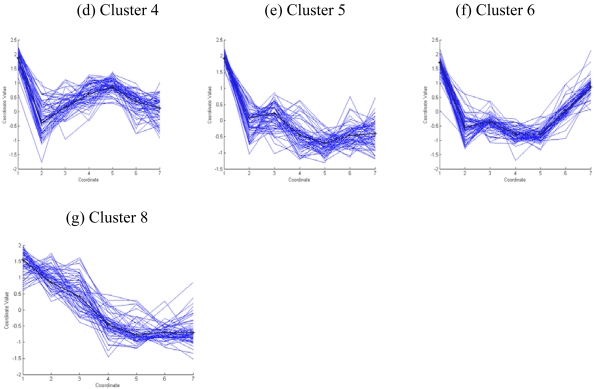
Cluster profile plots for clustering solution obtained by MODE-based clustering algorithm for yeast sporulation data.

In this section the best clustering solution provided by different algorithms on the sporulation data of yeast has been visualized using the cluster profile plot (in parallel coordinates) and the heatmap plot in MATLAB 7.0.4 version. Parallel coordinates [37] is a common way of visualizing high-dimensional geometry. A point in *n*-dimensional space is represented as a polyline with vertices on the parallel axes; the position of the vertex on the *i*-th axis corresponds to the *i*-th coordinate of the point. Cluster profile plots (in parallel coordinates) of seven clusters for the best clustering result (provided by MODE) on yeast sporulation data has been shown in Figure 10. The blue polylines indicate the member genes within a cluster while the black polyline indicates the centroid of that gene. Cluster profile plots (Figure 10) also demonstrate how the cluster profiles for the different groups of genes differ from each other, while the profiles within a group are reasonably similar.

**Figure 11.**
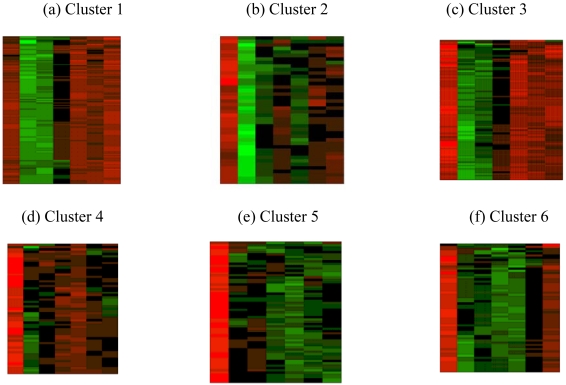

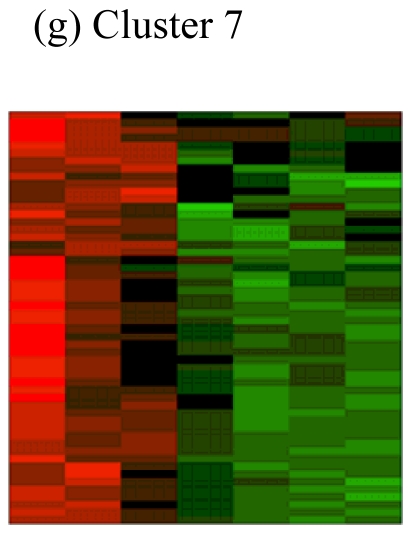
Heatmaps (Eisen plots) for clustering solution obtained by MODE-based clustering algorithm for yeast sporulation data.

**Figure 12.**
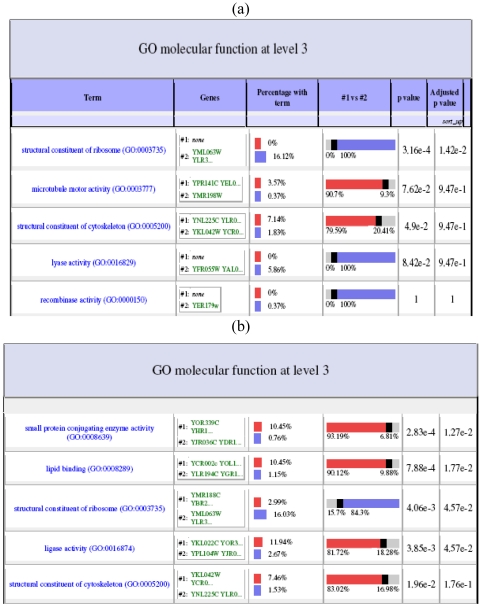
Part of FatiGO result for (a) cluster 6 and (b) cluster 2 of the best multi-objective clustering algorithm on yeast sporulation dataset.

In Heatmap (aka Eisen plot) [38], the expression value of a gene at a specific time point is represented by coloring the corresponding cell of the data matrix with a color similar to the original color of its spot on the microarray. The shades of red color represent higher expression level, the shades of green color represent low expression level and the colors towards black represent absence of differential expression values. In our representation, the genes are ordered before plotting so that the genes that belong to the same cluster are placed one after another. Figure 11 shows the Heatmap of the seven clusters generated by one run of the MODE algorithm for yeast sporulation data. It is evident from the figure that the expression profiles of the genes of a cluster are similar to each other and they produce similar colour patterns. Genes within the same cluster are expected to exhibit similar expressions as they should have similar functionality or contribute to the same biological processes. Here we attempt to determine the biological meanings of the clusters by using Gene Ontology (GO) terms using the popular web-based tool FatiGO [39] (www.fatigo.org) FatiGO extracts the GO terms for a query and a reference set of genes and further computes various statistics for the query set. In our experiment, a query is the set of genes of a cluster and union of the genes from the other clusters is taken as the reference set. The GO level is fixed at three. It is not possible to evaluate each cluster of the final solutions provided by all the algorithms here. So, two interesting clusters from the clustering results obtained on Yeast sporulation data set by the best performing algorithm (MODE in this case) is examined. Figure 12 shows a part of the FatiGO results of cluster 2 and 6 of multi-objective clustering on the sporulation data. It can be observed that the percentage of genes in the query cluster is considerably different from that of the reference cluster in almost all the functionalities. This implies that the correct genes are selected to remain in the same cluster.

## 5. Conclusions

This article compared the performances of two state-of-the-art multi-objective variants of DE with two other prominent multi-objective clustering algorithms. The test-suite included six hand-crafted and four real-life datasets including the gene expression data of budding yeast. The artificial datasets were chosen in two and three dimensions for the ease of visualization of clustering results and the number of clusters for them ranged from 3 to 9. The DE-variants and NSGA-II used the same objective functions based on the Xie-beni index and the FCM index. Tables 2 to 4 indicate that one or more multi-objective DE variants were always able to produce better final clustering solutions as compared to MOCK or NSGA II in terms of both adjusted Rand index and Silhouette index when all the algorithms were let run for an equal number of generations. Not only did they find out the correct partitions in the data but also in all cases they were able to determine an optimal number of classes with minimum standard deviations. Visualization of the yeast sporulation data clustering results with parallel coordinates and heatmap plots indicate that the MODE yielded compact and well separated clusters. Biological interpretations to the clustering solution have been given with the help of gene annotation using a web-based Gene Ontology tool (FatiGO). Experimental results indicate that DE holds immense promise as a candidate optimization technique for multi-objective clustering. Future research may extend the multi-objective DE-based clustering schemes to handle discrete chromosome representation schemes that no longer depend on cluster centroids and thus are not biased in any sense towards spherical clusters. As a scope of further research, the technique of multi-objective optimization with other cluster validity indices needs to be studied. Moreover, new ways of comparing the performance of multi-objective solutions have to be defined. The multi-objective clustering framework may be utilized for various real life applications, such as offline classification of sensor data, automatic image segmentation, document clustering etc.

## Figures and Tables

**Table 1.** Details of the datasets used.

**Dataset**	**Number of points**	**Number of clusters**	**Number of Characteristics**
Dataset_1	900	9	2
Dataset _2	76	3	2
Dataset _3	400	4	3
Dataset _4	300	6	2
Dataset _5	500	10	2
Dataset_ 6	810	3	2
Iris	150	3	4
Wine	178	3	13
Breast-Cancer	683	2	9
Yeast Sporulation	474	7	7

**Table 2.** Mean value of adjusted Rand index found and standard deviations (in parentheses) by four contestant algorithms over 30 independent runs on nine datasets.

**Dataset**	**Algorithms**							
	**MODE**		**DEMO**		**NSGA2**		**MOCK**	
	*k*	Adjusted Rand Index	*k*	Adjusted Rand Index	*k*	Adjusted Rand Index	*k*	Adjusted Rand Index
Dataset_1	**9.12 (1.46)**	**0.846199 (0.031257)**	9.43 (0.843)	0.828437 (0.046182)	9.37 (1.72)	0.802180 (0.004782)	8.52 (2.81)	0.810934 (0.0059348)
Dataset_2	**3.36 (0.65)**	**0.957621 (0.006312)**	3.74 (0.363)	0.9273464 (0.0008573)	3.16 (0.072)	0.9378123 (0.006821)	3.33 (1.03)	0.946547 (0.004536)
Dataset_3	4.14 (0.36)	0.951786 (0.004827)	**4.09 (0.24)**	**1.000000**	3.57 (0.51)	0.963841 (0.0046719)	3.78 (1.25)	0.878732 (0.0712523)
Dataset_4	**6.04 (0.25)**	**1.000000**	6.13 (1.27)	0.857463 (0.065639)	6.28 (0.46)	0.957818 (0.004678)	6.08 (0.51)	0.978761 (0.006734)
Dataset_5	9.24 (3.89)	0.983785 (0.076764)	**10.03 (0.37)**	**0.993173 (0.089371)**	12.43 (0.939)	0.947641 (0.006646)	10.41 (0.80)	0.9454568 (0.0012043)
Dataset_6	**5.19 (0.93)**	**0.93456 (0.08463)**	5.62 (0.867)	0.881136 (0.078348)	4.65 (1.58)	0.881395 (0.056483)	5.16 (0.38)	0.910294 (0.016743)
Iris	3.04 (0.16)	0.738626 (0.0756779)	**2.98 (0.40)**	**0.748784 (0.067457)**	2.16 (1.06)	0.715898 (0.005739)	3.05 (0.37)	0.736574 (0.075763)
Wine	**3.16 (0.46)**	**0.875849 (0.0087642)**	3.65 (0.83)	0.858876 (0.0035287)	3.88 (0.67)	0.828645 (0.0074653)	3.59 (0.46)	0.864764 (0.0034398)
Breast Cancer	**2.08 (0.38)**	**0.956456 (0.0056453)**	2.68 (0.64)	0.912173 (0.0043247)	2.57 (0.60)	0.944236 (0.006521)	2.10 (0.53)	0.9465731 (0.006748)

**Table 3.** Average Silhouette index and number of clusters found and standard deviations (in parentheses) by four contestant algorithms over 30 independent runs on the Yeast sporulation dataset.

**Dataset**	**Algorithms**							
	**MODE**		**DEMO**		**NSGA2**		**MOCK**	
	*k*	Silhouette Index	*k*	Silhouette Index	*k*	Silhouette Index	*k*	Silhouette Index
Yeast Sporulation	**7.08 (0.12)**	**0.676434 (0.00072)**	6.34 (0.32)	0.558619 (0.057832)	7.22 (0.68)	0.641306 (0.04813)	6.67 (0.857)	0.613567 (0.005738)

**Table 4.** Unpaired *t*-test Results for adjusted Rand index.

**Dataset**	**Std. Err**	***t***	**95% Conf. Intvl**	**Two-tailed *P***	**Statistical Significance Level**
Dataset_1	0.021	2.9201	-0.1050 to -0.0189	0.0059	**Very significant**
Dataset_2	0.013	5.0453	-0.0922 to -0.0394	< 0.0001	**Extremely Significant**
Dataset_3	0.002	17.965	-0.0452 to -0.0360	< 0.0001	**Extremely Significant**
Dataset_4	0.005	6.4431	-0.0419 to -0.0219	< 0.0001	**Extremely Significant**
Dataset_5	0.009	1.3744	-0.0309 to 0.0059	0.1774	Not Significant
Dataset_6	0.003	2.3999	-0.0118 to -0.0010	0.0214	**Significant**
Iris	0.009	6.3744	-0.0309 to 0.0059	0.1774	**Extremely Significant**
Wine	0.003	2.3999	-0.0118 to -0.0010	0.0278	**Significant**
Breast Cancer	0.009	1.3744	-0.0309 to 0.0059	0.1774	Not Significant

**Table 5.** Unpaired *t*-test results for Silhouette index

**Dataset**	**Std. Err**	***t***	**95% Conf. Intvl**	**Two-tailed *P*-Values**	**Statistical Significance Level**
Yeast Sporulation	0.003	2.3999	-0.0118 to -0.0010	0.0214	**significant**
